# Study on Early Pregnancy Diagnosis of Sows Based on Body Fluid Metabolite Detection Combined with Machine Learning Models

**DOI:** 10.3390/vetsci13050409

**Published:** 2026-04-22

**Authors:** Yun Feng, Ruonan Gao, Wengang Yang, Huiwen Lu, Weizeng Sun, Yun Zhang, Yujun Ren, Liming Gao, Mengxun Li, Qingchun Li, Guang Pu, Yongsheng Zhang, Zikai Ai, Kun Yan, Tao Huang

**Affiliations:** 1 College of Animal Science and Technology, Shihezi University, Shihezi 832003, China; 18785778959@163.com (Y.F.); zy13239178870@163.com (Y.Z.); renyujun@stu.shzu.edu.cn (Y.R.); mxli98@shzu.edu.cn (M.L.); 18899598025@163.com (Q.L.); pigfarmer_pu@163.com (G.P.); zhangyongsheng@shzu.edu.cn (Y.Z.); 16609933314@163.com (Z.A.); 18104603839@163.com (K.Y.); 2 Agricultural and Rural Bureau of Qitai County, Changji 831800, China; m13199933623@163.com; 3 Xinjiang Production and Construction Corps Seventh Division Animal Husbandry and Aquatic Products Development Service Center, Huyanghe 834034, China; 18999727216@163.com; 4 Xinjiang Pig Breeding Engineering Technology Research Center, Xinjiang Tecon Husbandry S&T Co., Ltd., Changji 831100, China; cankaoguanzhu@163.com (H.L.); 14799147803@163.com (W.S.); 5 Jinyi Farmers’ Breeding Cooperative, Huyanghe 834034, China; 13070499599@163.com

**Keywords:** sows, early pregnancy diagnosis, metabolites, chemical quantitative detection, machine learning

## Abstract

The conventional window for ultrasonic pregnancy diagnosis in sows is 22–25 days post-insemination, which often results in missed opportunities for the optimal re-insemination of non-pregnant sows and elevated production costs. This present study aimed to establish an early pregnancy detection method for sows at 12–18 days post-insemination, thereby providing a reference for efficient reproductive management. Saliva, urine and vaginal secretions were collected from sows during this period, and seven metabolites were quantified. Seven machine learning models were employed for data analysis, after which the optimal combination was determined, and the detection protocol was refined using recursive feature elimination. The results revealed that the majority of metabolites in saliva and urine differed significantly between pregnant and non-pregnant groups (*p* < 0.05). Among the models evaluated, the random forest algorithm exhibited the best predictive performance, with accuracy ranging from 0.59 to 1.00. Saliva sampled at 17 days post-insemination was identified as the optimal diagnostic medium, and 100% prediction accuracy was achieved by measuring only three metabolites: Glc, Ste, and Xan. The diagnostic approach established in this study allows pregnancy detection 5–8 days earlier than conventional methods, with the additional benefits of non-invasive sampling and minimal stress to sows. Accordingly, it provides a novel reference for enhancing the efficiency of swine production.

## Simple Summary

Determining pregnancy status in sows as early as possible following insemination is critically important for efficient pig production. Conventional ultrasonographic approaches typically enable pregnancy diagnosis at 22–25 days post-breeding, while current state-of-the-art equipment and techniques can achieve high diagnostic accuracy as early as 20 days after insemination. However, this prolonged waiting interval may lead to the loss of the optimal window for re-inseminating non-pregnant sows, thereby extending the non-productive days of sows and increasing overall production costs. The present study was designed to establish an earlier method for pregnancy detection in sows, specifically between 12 and 18 days post-breeding, to facilitate more efficient reproductive management in commercial swine operations. During this target period, saliva, urine, and vaginal secretions were collected from experimental sows, and seven distinct biochemical constituents were analyzed in these biofluids. Seven machine learning algorithms were subsequently applied to model the dataset for pregnancy classification, with the Random Forest model demonstrating the optimal predictive performance. The most accurate diagnostic outcome was achieved using saliva samples obtained at 17 days post-breeding, in which the measurement of only three analytes—glucose, steroids, and xanthine/hypoxanthine—yielded 100% diagnostic accuracy. This novel approach permits pregnancy detection 5–8 days earlier than traditional ultrasonography and employs non-invasive saliva sampling, thereby minimizing stress in sows. Collectively, this method enables early identification of pregnant sows and supports improvements in the efficiency and economic viability of swine farming.

## 1. Introduction

Pork constitutes the primary meat source for Chinese consumers. As a core sector of the agricultural economy, the sustainable and efficient development of the swine industry is essential for ensuring national food security and satisfying livelihood demands. In intensive pig production systems, the reproductive efficiency of breeding sows represents a key determinant of farm profitability, in which reducing non-productive days (NPD) and shortening the non-pregnant interval represent critical strategies for cost reduction and productivity improvement. Delayed confirmation of pregnancy status following insemination directly increases unnecessary feed expenditure, reduces pen utilization efficiency, and substantially diminishes the economic viability of swine production. Accordingly, the development of accurate and efficient early pregnancy diagnostic methods carries considerable practical significance and application potential for optimizing sow herd management and improving reproductive performance [1].

Currently, B-mode ultrasonography is widely implemented in intensive pig farms across China, with diagnostic accuracy exceeding 95%; however, its effective detection window is restricted to 22–25 days post-insemination. Marques et al. employed real-time ultrasonography and attained 100% accuracy for early pregnancy diagnosis as early as 20 days after insemination [2]. Since the estrous cycle of non-pregnant sows is approximately 21 days, such approaches frequently result in missed opportunities for timely re-insemination and are unable to satisfy the requirements for efficient reproductive management of non-pregnant sows. Other conventional methods, including boar exposure testing, radioimmunoassay, and similar techniques, fail to fully satisfy the technical demands of intensive pig production, owing to limitations such as low diagnostic accuracy, cumbersome operation procedures, high analytical costs, and pronounced stress responses in sows [3]. In traditional chemical molecular diagnostic approaches, pregnancy in sows can be diagnosed by quantifying the concentrations of estrone sulfate and progesterone in peripheral blood as early as 20 days post-insemination. However, this method is highly invasive to sows and relies on either radioimmunoassay or enzyme-linked immunosorbent assay techniques, which results in relatively high detection costs [4]. Accordingly, there is an urgent need in the swine industry for an early pregnancy diagnosis protocol that is easy to operate, cost-controllable, highly sensitive, and minimally stressful to sows. Our research group has previously identified seven categories of characteristic differential metabolites in saliva, urine, and vaginal secretion samples from pregnant versus non-pregnant sows, including Phenols (Pheo), Phosphates (Pho), Primary amines (Ami), Steroids (Ste), Xanthine/Hypoxanthine (Xan), Flavonoids (Fla), and Glucose (Glc). The physiological functions of these metabolites are closely associated with metabolic reprogramming in sows during pregnancy: steroids, as precursors of sex hormones, exhibit concentration changes that directly reflect fluctuations in pregnancy-related hormone levels [5,6]; glucose, a core substrate for energy metabolism in pregnant sows, has a significantly increased demand as embryonic development progresses [7]; xanthine/hypoxanthine are involved in purine metabolism and can reflect the status of cell proliferation and oxidative stress during pregnancy [8,9]; phenols, phosphates, primary amines, and flavonoids indirectly contribute to pregnancy maintenance by regulating antioxidant balance, bone metabolism, protein synthesis, and immune function, respectively [10,11,12,13,14,15]. Collectively, these characteristics provide a physiological basis for their potential application as pregnancy diagnostic biomarkers.

Based on the chemical properties of the seven aforementioned metabolite categories, this present study aimed to quantitatively analyze target metabolites in three biofluid samples and construct a multi-dimensional pregnancy diagnosis model by integrating seven machine learning algorithms, including Multilayer Perceptron (MLP), Random Forest (RF), Naive Bayes (NB), Logistic Regression (LR), Fisher Linear Discrimination (FLD), Support Vector Machine (SVM) and Extreme Gradient Boosting (XGBoost, XGB). By validating and comparing the classification performance of these models—including Matthews Correlation Coefficient, AUC, cross-entropy loss, and Brier score—the optimal diagnostic protocol was identified, characterized by the earliest detectable timepoint, the minimum number of feature parameters, and superior predictive performance. The overall objective of this study was to develop an efficient and cost-effective technical system for the early pregnancy diagnosis of sows, as well as to provide scientific evidence for investigating the metabolic regulatory mechanisms of early pregnancy and innovating diagnostic technologies in intensive swine production.

Conventional ultrasonographic detection methods have a relatively delayed detection window, which may result in the loss of opportunities for rebreeding non-pregnant sows. In contrast, traditional chemical diagnostic approaches—such as those targeting progesterone and estrone sulfate—are invasive and cumbersome to implement. Therefore, the present study sought to apply biofluid metabolite detection combined with seven machine learning algorithms to the early pregnancy diagnosis of sows, thereby overcoming the limitations of conventional methods. The study aimed to develop an earlier, non-invasive diagnostic method for early pregnancy and provide scientific support for the sustainable development of the swine industry.

## 2. Materials and Methods

### 2.1. Experimental Material

#### 2.1.1. Treatment of Experimental Animals

All experimental animals used in this study were provided by Xinjiang Tecon Animal Husbandry Technology Co., Ltd., including 79 Landrace × Yorkshire crossbred replacement gilts (age: 225–250 days; body weight: 135–150 kg) with good body condition. All gilts were reared under consistent environmental conditions, with standardized feeding and housing management protocols. All animals were in good health, free from genetic diseases and physical deformities. The pig house environment was maintained at a temperature of 20–25 °C and a relative humidity of 50–55%. The production system adopted a batch-wise, all-in-all-out management model, and the gilts were fed a commercial compound feed. All gilts were subjected to estrus synchronization treatment, with the specific protocol as follows: All gilts were continuously administered altrenogest via oral gavage for 18 days. Twenty-four hours after altrenogest withdrawal, each gilt was intramuscularly injected with 1000 IU of serum gonadotropin. Three days later, each gilt was intramuscularly injected with 2 mL of gonadorelin injection. Following the onset of estrus, the gilts were artificially inseminated in batches using the following procedure: a semen catheter was inserted into the gilt’s vagina at a 45° upward angle until the catheter tip was engaged by the cervix and could no longer advance. The semen bottle was then hung upside down above the gilt’s back to allow spontaneous semen flow. On days 23–25 post-insemination, pregnancy diagnosis was performed using a Gandalf GDF-A4 ultrasonic pregnancy detector (Manufactured by Zhengzhou Gandor Electronic Technology Co., Ltd., Zhengzhou, Henan Province, China) specifically designed for swine.

#### 2.1.2. Sample Collection and Pretreatment

From 12 to 18 days post-insemination, saliva, urine, and vaginal secretion samples were collected as follows: a sterile gauze pad was attached to one end of the copper wire, and saliva samples were collected prior to daily feeding by encouraging gilts to chew on the sterile gauze. Midstream urine samples were collected using clean, disposable paper cups. Vaginal secretions were collected by inserting a sterile cotton swab 15–20 cm into the gilt’s vagina, allowing it to remain in place for 30 min before retrieval. A total of 40–50 samples each of saliva, urine, and vaginal secretions were collected from pregnant gilts (P group), while 30–40 samples each of the same biofluids were obtained from non-pregnant gilts (NP group) (Table 1). All collected samples were transferred to 5 mL centrifuge tubes, snap-frozen in liquid nitrogen for immediate preservation, transported back to the laboratory, and subsequently stored long-term at −80 °C (−80 °C Freezer: Thermo Scientific, Marietta, OH, USA). Prior to experimental analysis, all samples were pretreated: they were centrifuged at 10,000 rpm for 1 min to remove solid impurities (High-Speed Refrigerated Centrifuge: Thermo Scientific, Marietta, OH, USA), and the resulting supernatant was collected for subsequent analyses.

Given that vaginal secretions were collected using sterile cotton swabs, a dedicated extraction protocol was required: cotton swabs saturated with vaginal secretions were transferred to 10 mL centrifuge tubes, followed by the addition of 7 mL of 60% anhydrous ethanol (Tianjin Damao Chemical Reagent Factory, Tianjin, China). After thorough shaking and mixing, the tubes were incubated overnight at 4 °C. On the following day, the mixture was centrifuged at 10,000 rpm for 1 min to remove solid contaminants, and the resulting supernatant was collected for subsequent analyses.

### 2.2. Detection of Metabolites in Body Fluid Samples

In this study, the concentrations of seven categories of chemical substances in three bodily fluids were determined via the following methods. Standard curves relating the concentration of each category of substances to absorbance were established respectively (Figure S1). In addition, curves illustrating the effects of soaking time in 60% anhydrous ethanol on the extraction efficiency of various chemical substances in vaginal secretions were plotted (Figure S2). Ultimately, overnight soaking (more than 3 h) at 4 °C was identified as the optimal protocol.

#### 2.2.1. Detection of Phenols

① Preparation of phenol standard solution: Precisely weigh 0.05 g of phenol (purity ≥99.5%, analytical grade: Shanghai Macklin Biochemical Technology Co., Ltd., Shanghai, China), add 1.25 mL of 0.2 N sodium hydroxide (Flake Sodium Hydroxide: Shanghai Wokai Biotechnology Co., Ltd., Shanghai, China) solution, mix thoroughly, and transfer to a 50 mL volumetric flask. Dilute to the graduation mark with distilled water to obtain the phenol stock solution. For use, pipette 0.5 mL of the phenol stock solution into a 50 mL volumetric flask, dilute to the graduation mark with distilled water to prepare the 10 μg/mL phenol standard working solution. Subsequently, perform gradient dilution of the working solution to obtain a series of standard solutions at concentrations of 0, 1, 2, 4, 6, and 7 μg/mL according to experimental requirements, and store under refrigeration for later use.
② Preparation of chromogenic reagent: Precisely weigh 0.025 g of 2,6-dichloroquinone-4-chloroimide powder (Shanghai Macklin Biochemical Technology Co., Ltd., Shanghai, China), add 10 mL of anhydrous ethanol for ultrasonic dissolution, then transfer the solution to a 50 mL volumetric flask and dilute to the graduation mark with anhydrous ethanol to obtain a 0.05% (*w*/*v*) 2,6-dichloroquinone-4-chloroimide chromogenic reagent. Store the reagent at 4 °C in a refrigerator (Thermo Scientific, Marietta, OH, USA) away from light for later use.
③ Preparation of boric acid-potassium chloride buffer solution: Precisely weigh 0.155 g of boric acid (Tianjin Yongsheng Fine Chemical Co., Ltd., Tianjin, China) and 0.175 g of potassium chloride (Tianjin Sheng’ao Chemical Reagent Co., Ltd., Tianjin, China), dissolve in an appropriate amount of distilled water, then add 1.6 mL of 1 N sodium hydroxide solution to adjust the pH to 9.0. Transfer the solution to a 50 mL volumetric flask and dilute to the graduation mark with distilled water to obtain the boric acid-potassium chloride buffer solution for subsequent use.
④ Add 100 μL of the sample to be tested, 100 μL of boric acid-potassium chloride buffer solution and 20 μL of 2,6-dichloroquinone-4-chloroimide chromogenic reagent sequentially into the wells of the microplate, mix gently with shaking, and then incubate at room temperature for 30 min to allow the reaction to proceed.
⑤ The absorbance value of each well was measured at a wavelength of 630 nm using a microplate reader (Thermo Scientific, Marietta, OH, USA).

#### 2.2.2. Detection of Phosphates

① Perform gradient dilution according to the concentration differences in phosphate in different body fluids to be tested: saliva was diluted 40-fold, urine 100-fold, and the 60% anhydrous ethanol extract of vaginal secretions 20-fold.
② Preparation of phosphate standard working solution: Pipette 10 μL of 5 mM phosphate standard solution, add 990 μL of distilled water, and make up the volume to 1 mL to obtain a 50 μM phosphate working solution. Store at 4 °C under refrigeration for later use (Phosphate Detection Kit: Beyotime Biotechnology Co., Ltd., Shanghai, China).
③ Preparation of chromogenic reagent: Mix Malachite Green Reagent A and Malachite Green Reagent B thoroughly at a volume ratio of 2:1. Prepare fresh and use immediately (70 μL of chromogenic reagent is required per sample; prepare on demand).
④ Add 200 μL of the sample to be tested into each microplate well, followed by 70 μL of chromogenic reagent. Mix gently with shaking and incubate at room temperature for 30 min to allow the reaction to proceed.
⑤ Measure the absorbance of each well at a wavelength of 630 nm using a microplate reader.

#### 2.2.3. Detection of Primary Amines

① Preparation of primary amines standard solution: Take the 50 μg/mL primary amines standard stock solution, allow it to equilibrate to room temperature, and dilute it to 10 μg/mL with distilled water. Further perform gradient dilution to prepare a series of standard working solutions at concentrations of 0, 1, 2, 4, 6 and 7 μg/mL for subsequent use (Ninhydrin Kit: Regen Biotechnology Co., Ltd., Beijing, China).
② Preparation of ninhydrin stock solution and working solution: Add 100 mg of ninhydrin powder to 10 mL of ninhydrin diluent, shake until completely dissolved to obtain the ninhydrin stock solution. Mix an appropriate volume of ninhydrin stock solution with AA Assay buffer at a ratio of 35:3 to prepare the ninhydrin working solution (45 μL required per sample, prepare on demand), and prepare fresh for immediate use.
③ Preparation of vitamin C working solution: Weigh an aliquot of vitamin C powder, dissolve it in 10 mL of distilled water, mix thoroughly, and store at 4 °C under refrigeration for later use.
④ Transfer to a 1.5 mL centrifuge tube, add 30 μL of the sample to be tested, 45 μL of the ninhydrin working solution and 15 μL of vitamin C working solution, mix well, and incubate in a water bath at 80 °C for 20 min.
⑤ Immediately transfer to an ice bath for cooling after removal, add 210 μL of 60% anhydrous ethanol, and mix thoroughly.
⑥ Transfer 300 μL of the reacted solution to a microplate and measure its absorbance at a wavelength of 570 nm.

#### 2.2.4. Detection of Steroids

① Preparation of steroid standard solution: Pipette 20 μL of the 5 mM steroid standard solution, add 180 μL of assay buffer, mix thoroughly to prepare a 500 μM steroid standard solution. Separately take 0, 1, 2, 3, 5, 10, 20 and 50 μL of the 500 μM steroid standard solution, and dilute with an appropriate volume of assay buffer to prepare steroid standard solutions with concentrations of 0, 10, 20, 30, 50, 100, 200 and 500 μM for subsequent use (Cholesterol Detection Kit: Beyotime Biotechnology Co., Ltd., Shanghai, China).
② Preparation of steroid assay working solution: According to the dosage of 50 μL per sample, mix assay buffer, Amplex Red, cholesterol esterase and enzyme mixture thoroughly at a volume ratio of 22:1:1:1 for subsequent use.
③ Add 50 μL of the sample to be tested into each microplate well, followed by 50 μL of the assay working solution, and incubate at 37 °C away from light for 30 min.
④ Measure the absorbance at a wavelength of 570 nm.

#### 2.2.5. Detection of Xanthine/Hypoxanthine

① Preparation of xanthine standard solution: Pipette 2 μL of the 50 mM xanthine standard solution, add 198 μL of xanthine assay buffer, mix thoroughly to prepare a 500 μM xanthine standard solution. Subsequently, pipette 0, 1, 2, 3, 5, 10, 20 and 50 μL of the 500 μM xanthine standard solution separately, add an appropriate volume of xanthine assay buffer to make up the volume to 50 μL, yielding xanthine standard solutions with concentrations of 0, 10, 20, 30, 50, 100, 200 and 500 μM, respectively, for subsequent use (Xanthine/Hypoxanthine Detection Kit: Beyotime Biotechnology Co., Ltd., Shanghai, China).
② Preparation of xanthine assay working solution: Prepare the working solution at a volume ratio of 22:1:1:1 with xanthine assay buffer, Amplex Red, xanthine oxidase and Xanthine Developer, based on a dosage of 50 μL per sample, and store for subsequent use.
③ Add 50 μL of the sample to be tested into each microplate well, then add 50 μL of the assay working solution, and incubate at 37 °C in the dark for 30 min.
④ Measure the absorbance at a wavelength of 570 nm.

#### 2.2.6. Detection of Flavonoids

① Preparation of flavonoid standard solutions: Pipette 0, 24, 48, 72, 96, 120, 144 and 180 μL of the flavonoid standard solution separately, add an appropriate volume of anhydrous ethanol to make up the volume to 1200 μL, yielding flavonoid standard solutions with concentrations of 0, 20, 40, 60, 80, 100, 120 and 150 μg/mL, respectively, for subsequent use (Flavonoid Detection Kit: Wuhan Elabscience Biotechnology Co., Ltd., Wuhan, China).
② Add 75 μL of the sample solution to each microplate well, add 10 μL of nitrite solution, shake for 5 s, and stand at room temperature for 5 min; add 30 μL of aluminon solution, shake for 5 s, and stand at room temperature for 5 min; add 180 μL of alkaline solution, shake for 5 s, and stand at room temperature for reaction for 15 min.
③ Measure the absorbance at a wavelength of 510 nm.

#### 2.2.7. Detection of Glucose

① Preparation of glucose standard solution: Pipette 0, 0.25, 0.5, 1, 2.5, 5, 10 and 20 μL of the 200 mg/mL glucose standard solution separately, add distilled water to make up the volume to 1 mL, yielding glucose standard solutions with concentrations of 0, 5, 10, 20, 50, 100, 200 and 400 mg/dL respectively, for subsequent use (Glucose Detection Kit: Beyotime Biotechnology Co., Ltd., Shanghai, China).
② Add 20 μL of the sample to be tested into a 200 μL PCR tube, followed by 170 μL of o-toluidine assay reagent, and incubate at 95 °C for 8 min in a PCR thermal cycler.
③ After completion of the reaction, allow the temperature to cool down to 4 °C, transfer 180 μL of the solution from the PCR tube to a microplate, and measure its absorbance at a wavelength of 630 nm.

### 2.3. Construction and Feature Screening of Machine Learning Models

After acquiring the concentration data of the seven types of metabolites in the three biofluids, a classification dataset was constructed, comprising non-pregnant gilts and gilts at 12–18 days of gestation. Specifically, the detection results of the corresponding biofluids from the two groups were integrated separately and labeled with group identifiers. First, the concentration data of the seven metabolites were standardized using the z-score method to eliminate dimension effects. Subsequently, to objectively evaluate the predictive performance of the machine learning model, all data were randomly split into a training set and a test set at a 7:3 ratio. The training set was used to construct and train the model, while the test set was independently employed to assess the actual prediction performance of the trained models on unknown gilt samples, thereby avoiding model overfitting. The training set teaches the model to recognize pregnancy-related patterns, while the test set evaluates how well the model works on unseen samples, avoiding over-optimism (overfitting).

Subsequently, seven machine learning models were constructed for the fitting and prediction of gilt pregnancy status, including Multilayer Perceptron (MLP), Random Forest (RF), Naive Bayes (NB), Logistic Regression (LR), Fisher Linear Discriminant (FLD), Support Vector Machine (SVM), and Extreme Gradient Boosting (XGB). The hyperparameter settings of each model are detailed in Table 2.

The Matthews Correlation Coefficient (MCC), Area Under the Receiver Operating Characteristic Curve (ROC-AUC), Logarithmic Loss (Logloss), and Brier Score were employed to evaluate the fitting accuracy and predictive performance of each model on the test set. Additionally, the reliability of the model prediction results was assessed by plotting the predicted probabilities distribution curve of the test set samples.

The predictive accuracy of pregnancy status in gilts was compared across different biofluid types, different gestational ages (12–18 days post-insemination), and different machine learning models, with the aim of screening out the optimal combination of biofluid × gestational age × machine learning model for gilt pregnancy diagnosis.

**Figure 1:**
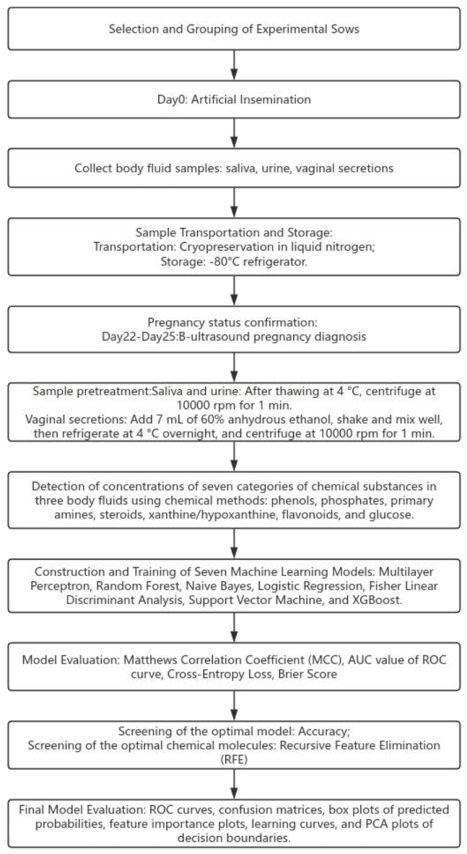
Flowchart of Experimental Procedures.

For the selected optimal model, the mean impurity decrease (from the RF) and SHAP (SHapley Additive exPlanations) methods were employed to calculate the feature importance of the seven metabolites. Additionally, the recursive feature elimination (RFE) method was used to screen for the optimal feature combination that achieves the highest pregnancy diagnosis accuracy with the minimum number of metabolites. The experimental procedure of this study is shown in Figure 1.

Note on the use of Generative Artificial Intelligence (GenAI): The Python 3.13.5 code used for data preprocessing, machine learning model construction, model training, performance evaluation, and feature importance analysis in this section was initially generated with the assistance of Doubao and Kimi. All GenAI-generated code was manually verified, debugged, and modified by the authors to ensure its accuracy, reproducibility, and consistency with the study’s research objectives. The authors bear full responsibility for the final version of the code and the analytical results derived from it. The complete and validated Python code has been included in the Supplementary Materials for review.

## 3. Results

### 3.1. Differences in the Concentration Variation Trends of Various Metabolites in Different Biofluids of Gilts During Early Pregnancy

The concentration variation trends of the various metabolites differ across the different biofluids of gilts.

**Figure 2:**
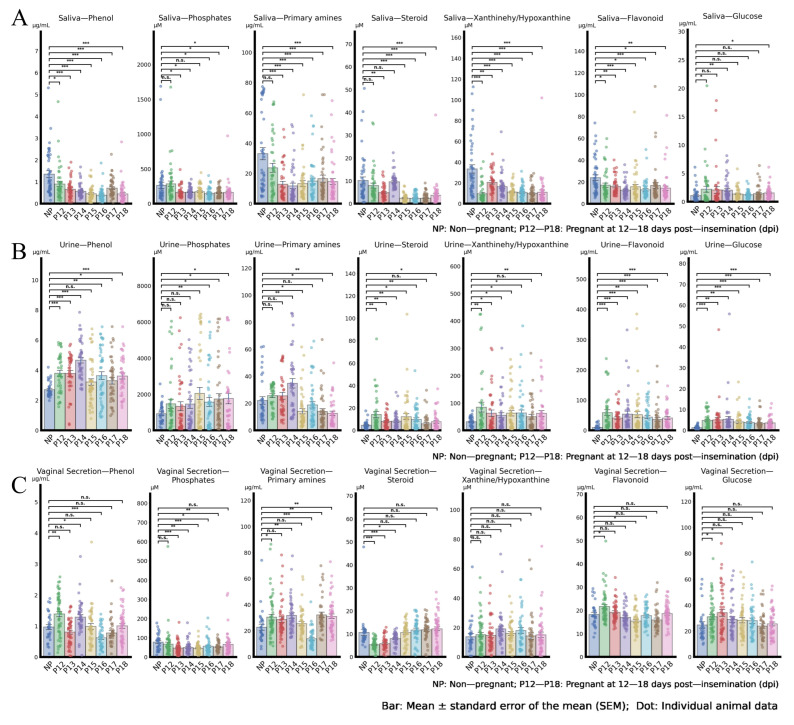
Dynamic changes in concentrations of seven metabolites in three body fluids of sows from 12 to 18 days post-insemination (dpi). (**A**) Saliva metabolite concentrations; (**B**) Urine metabolite concentrations; (**C**) Vaginal secretion concentrations. The X-axis indicates the experimental groups: NP = non-pregnant sows, P12–P18 = pregnant sows at 12, 13, 14, 15, 16, 17, and 18 dpi, respectively. (***: *p* < 0.001; **: *p* < 0.01; *: *p* < 0.05; n.s.: *p* ≥ 0.05).

Figure 2 shows the dynamic changes in the concentrations of seven metabolite categories (phenols, phosphates, primary amines, steroids, xanthine-hypoxanthine, flavonoids, and glucose) in sow saliva, urine, and vaginal secretions from 12 to 18 days post-insemination (dpi). The X-axis represents the experimental groups, with NP indicating non-pregnant sows and P12–P18 indicating pregnant sows at 12 to 18 dpi. Bars represent the mean ± standard error of the mean (SEM), and scattered dots show individual animal data. As shown in Figure 2A, most metabolite categories in saliva (including phenols, phosphates, primary amines, steroids, xanthine-hypoxanthine, and flavonoids) exhibited significantly lower concentrations in pregnant sows than in non-pregnant sows at multiple time points (*p* < 0.05, *p* < 0.01, or *p* < 0.001). In contrast, urine and vaginal secretion metabolites showed less consistent differences between pregnant and non-pregnant sows (Figure 2B,C), with only partial categories showing significant changes. These results indicate that saliva metabolites have the most potential as early pregnancy diagnostic biomarkers in sows. Biologically, the marked elevation of phenols, xanthine-hypoxanthine, and glucose in saliva likely reflects the increased metabolic demands of the developing conceptus and the maternal endocrine shift that occurs during implantation (days 12–18 in pigs). Saliva, as an ultrafiltrate of blood, captures these systemic changes non-invasively, whereas urine and vaginal secretions are more influenced by local urogenital flora and dilution effects, explaining their less consistent patterns.

In saliva, the concentrations of phenols and xanthine/hypoxanthine differed significantly between pregnant and non-pregnant gilt samples at 12 days post-insemination (*p* < 0.05), and these concentration differences reached an extremely significant level from 13 days post-insemination onwards (*p* < 0.01), xanthine and hypoxanthine are purine metabolites; their increase may indicate enhanced nucleotide turnover in the rapidly growing trophoblast, or a mild hypoxic state during embryo attachment. The other metabolites exhibit similar variation trends. Additionally, except for glucose, steroids, flavonoids, phosphates, and primary amines were significantly downregulated in the saliva of pregnant gilts (*p* < 0.05) (Figure 2A).

In urine, flavonoids and glucose exhibited significant concentration differences between pregnant and non-pregnant groups (*p* < 0.05), and both were significantly upregulated in the urine of pregnant gilts (*p* < 0.05). Phenols, phosphates, xanthine/hypoxanthine, and other metabolites all showed varying degrees of upward trends. Among these, phosphates exhibited no significant variation from 12 to 14 days post-insemination (*p* > 0.05) and only displayed a significant upward trend after day 15 (*p* < 0.05). The concentrations of phenols, primary amines, and xanthine/hypoxanthine increased continuously from 12 to 14 days post-insemination and gradually stabilized after day 15 (Figure 2B).

In vaginal secretions, most metabolites exhibited minimal significant changes in concentration between pregnant and non-pregnant sow samples; only phosphates, primary amines, phenols, and steroids showed significant concentration differences at specific gestational ages (*p* < 0.05). In contrast, xanthine/hypoxanthine showed no significant differences in concentration between pregnant and non-pregnant samples across different gestational days (*p* > 0.05). Additionally, four metabolites in vaginal secretions—phenols, steroids, flavonoids, and glucose—exhibited obvious concentration fluctuations from 12 to 14 days post-insemination, and their concentrations gradually stabilized after day 15 (Figure 2C) (Table 3).

### 3.2. Performance Differences in Different Machine Learning Models in Pregnancy Diagnosis

#### 3.2.1. Evaluation of Model Performance in Different Samples

**Figure 3:**
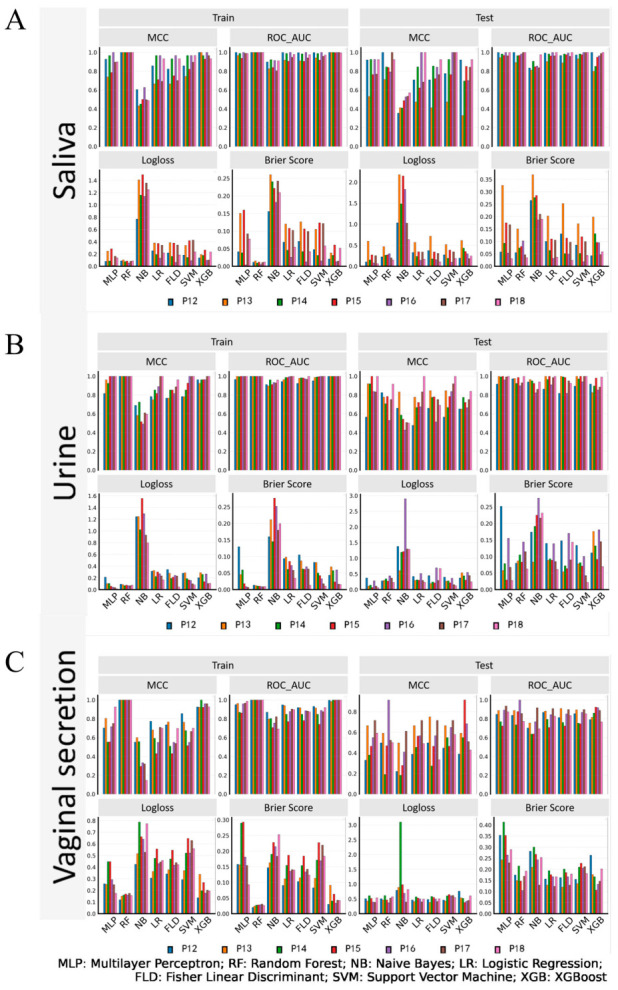
Performance evaluation of seven machine learning models for sow pregnancy prediction on training and test sets across 12–18 days post-insemination (dpi). (**A**) Saliva samples; (**B**) Urine samples; (**C**) Vaginal secretion samples. For each body fluid, models were evaluated using four metrics: MCC (Matthews Correlation Coefficient), ROC_AUC (Area Under the Receiver Operating Characteristic Curve), Logloss (Logarithmic Loss), and Brier Score. The X-axis represents the seven machine learning models: MLP (Multilayer Perceptron), RF (Random Forest), NB (Naive Bayes), LR (Logistic Regression), FLD (Fisher Linear Discriminant), SVM (Support Vector Machine), and XGB (XGBoost). Bar colors indicate different time points: P12–P18 = pregnant sows at 12, 13, 14, 15, 16, 17, and 18 dpi, respectively. Non-pregnant (NP) sows were included as the reference group for model training and testing at all time points. Higher MCC and ROC_AUC values, and lower Logloss and Brier Score values, indicate better model performance.

Figure 3 shows the performance of seven machine learning models (MLP, RF, NB, LR, FLD, SVM, XGB) for early pregnancy diagnosis in sows, evaluated on both training and test sets across 12–18 dpi. For each model, four metrics were used to assess performance: MCC (Matthews Correlation Coefficient), ROC_AUC (Area Under the Receiver Operating Characteristic Curve), Logloss, and Brier Score. Higher MCC and ROC_AUC values, and lower Logloss and Brier Score values, represent better predictive performance. As shown in Figure 3A, the RF model achieved the most stable and excellent performance in saliva samples, with near-perfect MCC and ROC_AUC values (close to 1.0) and extremely low Logloss and Brier Score values across all time points, outperforming all other models. In contrast, urine and vaginal secretion models (Figure 3B,C) showed lower and less stable performance, with higher Logloss and Brier Score values, confirming that saliva is the optimal body fluid for early pregnancy diagnosis. From a physiological perspective, saliva reflects the composite endocrine and metabolic status of the sow without interference from urinary tract contaminants or vaginal flora. The high AUC and MCC values for saliva-based models suggest that early pregnancy induces a consistent, systemic biochemical signature that machine learning algorithms can readily capture.

In saliva, most machine learning models exhibited excellent performance. Among these, the RF model demonstrated outstanding fitting ability in the training sets of samples at different gestational ages, with both the MCC and ROC-AUC reaching 1.0000; the Logloss values for samples at all gestational ages were below 0.1036, and the Brier Score values were all below 0.0153. In the test sets, the MCC values for samples at different gestational ages ranged from 0.7125 to 1.0000, the ROC-AUC values ranged from 0.8916 to 1.0000, the Logloss values ranged from 0.1483 to 0.4673, and the Brier Score values ranged from 0.0346 to 0.1507. Furthermore, model performance showed a gradual improvement trend with increasing gestational age. Other models, including MLP, LR, FLD, SVM, and XGB, also exhibited excellent fitting ability and predictive performance, whereas only the NB model showed relatively poor overall performance (Figure 3A).

In urine, the machine learning models exhibited a performance trend similar to that observed in saliva. The RF model and most other models performed well in both the training and test sets, demonstrating strong ability to fit the training set data and accurately predict the test set data. Among these, the RF model exhibited the best overall performance. In the training sets of samples at different gestational ages, both MCC and ROC-AUC reached 1.0000, Logloss ranged from 0.0668 to 0.0912, and the Brier Score ranged from 0.0086 to 0.0132. In the test sets, the RF model’s MCC values for samples at different gestational ages ranged from 0.5290 to 0.9167, and its ROC-AUC values ranged from 0.8994 to 1.0000, with model performance gradually improving as gestational age increased. Other models, including MLP, LR, FLD, SVM, and XGB, also demonstrated excellent fitting ability and predictive performance, whereas the NB model exhibited relatively unsatisfactory overall performance (Figure 3B).

In vaginal secretions, the various machine learning models exhibited considerable performance fluctuations across samples at different gestational ages. Among these, the RF model achieved the most stable overall performance: in the training set, both the MCC and ROC-AUC reached 1.0000 for samples at all gestational ages, with Logloss ranging from 0.1193 to 0.1755 and the Brier Score ranging from 0.0200 to 0.0305; in the test set, MCC values ranged from 0.1890 to 0.9129, ROC-AUC values ranged from 0.7355 to 1.0000, Logloss values ranged from 0.3700 to 0.6111, and Brier Score values ranged from 0.1046 to 0.2148. Among the other models, XGB also exhibited relatively stable fitting ability and predictive performance. Although the MLP model showed relative stability in the other three metrics, it had the highest Brier Score among all models, with Brier Score values ranging from 0.0928 to 0.2930 in the training set and from 0.2292 to 0.4132 in the test set (Figure 3C).

#### 3.2.2. Distribution of Model Prediction Probabilities in Different Samples

The reliability of the models in predicting pregnant and non-pregnant samples can be evaluated by examining the distribution of predicted probabilities across the different models.

**Figure 4:**
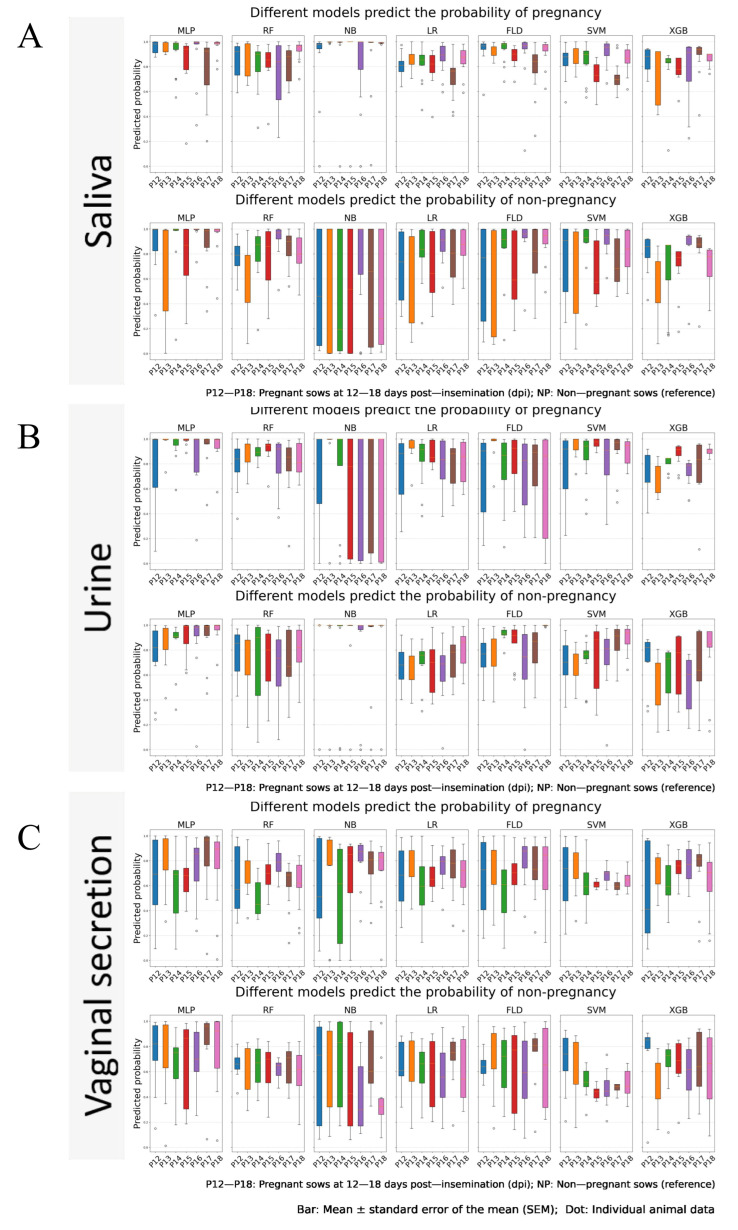
Predicted probability distribution for pregnancy and non-pregnancy status by seven machine learning models in the test set, across three body fluids. (**A**) Saliva samples; (**B**) Urine samples; (**C**) Vaginal secretion samples. For each body fluid, the upper row shows predicted pregnancy probabilities for pregnant sow samples, and the lower row shows predicted non-pregnancy probabilities for non-pregnant sow samples. The X-axis represents time points: P12–P18 = pregnant sows at 12, 13, 14, 15, 16, 17, and 18 days post-insemination (dpi), respectively, with non-pregnant (NP) sows included as the reference group. The Y-axis indicates the predicted probability (range: 0–1, where 1 = high confidence in the predicted status). Box plots show the distribution of predicted probabilities, and scattered dots represent individual sample data. The seven machine learning models are: MLP (Multilayer Perceptron), RF (Random Forest), NB (Naive Bayes), LR (Logistic Regression), FLD (Fisher Linear Discriminant), SVM (Support Vector Machine), and XGB (XGBoost).

Figure 4 shows the predicted probability distributions for pregnancy and non-pregnancy status generated by seven machine learning models in the test set, across saliva, urine, and vaginal secretion samples. For each body fluid, the upper row displays the predicted pregnancy probabilities for confirmed pregnant sows at 12–18 dpi, while the lower row displays the predicted non-pregnancy probabilities for confirmed non-pregnant sows. A higher predicted probability (closer to 1) indicates greater model confidence in the correct classification.

In saliva, there were significant differences in the predictive reliability of different models for distinguishing between pregnant and non-pregnant samples. For instance, the MLP, NB, LR, FLD, and SVM models demonstrated high reliability in predicting pregnant samples. However, their reliability in predicting non-pregnant samples was relatively low, with a high risk of misclassification. In contrast, the RF and XGB models exhibited balanced and relatively reliable predictive performance for both pregnant and non-pregnant samples (Figure 4A). From a diagnostic biology perspective, a model that reliably distinguishes both pregnant and non-pregnant samples with high confidence (probabilities near 0 or 1) is essential for farm decision-making. The balanced performance of RF and XGB in saliva indicates that the systemic metabolic signature of early pregnancy is sufficiently robust to be captured without bias toward one class.

In urine samples, different machine learning models exhibited significant variations in predictive performance across different gestational ages. The NB and FLD models demonstrated high predictive reliability for non-pregnant samples but weak predictive performance for pregnant samples. In contrast, the RF, LR, SVM, and XGB models showed good predictive reliability for pregnant samples yet low predictive reliability for non-pregnant samples. The MLP model achieved favorable predictive reliability for both pregnant and non-pregnant urine samples (Figure 4B).

In vaginal secretion samples, all machine learning models exhibited significant fluctuations in the predicted probabilities for pregnant and non-pregnant samples, accompanied by low predictive reliability and a high risk of misclassification (Figure 4C).

### 3.3. Multidimensional Screening Identifies the Optimal Diagnostic Combination: Saliva-Day 17-RF

**Figure 5:**
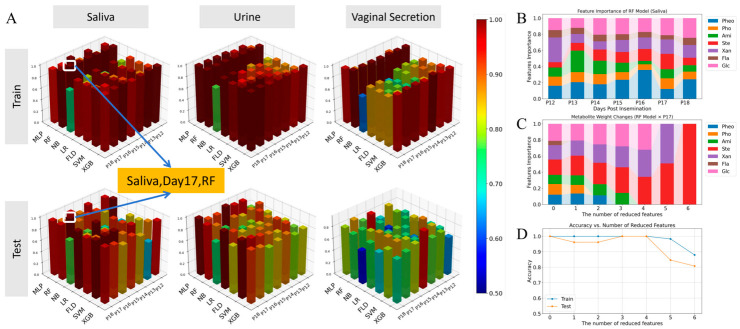
Optimal combination screening for pregnancy diagnosis and core metabolite identification. (**A**) Three-dimensional performance comparison across different body fluids (Saliva, Urine, Vaginal Secretion), time points (12–18 days post-insemination, dpi), and machine learning models. The height and color of the 3D bars represent model accuracy (higher values indicate better performance), with the optimal combination (Saliva, Day 17, RF) highlighted (**B**). Feature Importance variations in the RF Model for Sow Saliva at Different Days Post-Insemination (P12–P18). The color gradient indicates the importance score (0 = low, 1 = high). (**C**) Changes in the weight of seven metabolite categories during Sequential feature reduction. (**D**) Changes in model accuracy as metabolites are removed sequentially. The X-axis in (**B**) represents 12–18 dpi; the X-axes in (**C**,**D**) represent the number of metabolites removed (1–6). Metabolite abbreviations: Phe = Phenols, Pho = Phosphates, Ami = Primary Amines, Ste = Steroids, Xan = Xanthine-Hypoxanthine, Fla = Flavonoids, Glc = Glucose.

By comparing the prediction accuracy of sow pregnancy status across different sample types, post-insemination days, and model types, combined with the MCC, ROC-AUC, Logloss, and Brier Score of prediction models under different combinations, as well as the predictive reliability of the models for test set samples, the optimal prediction combination was screened out. Figure 5 summarizes the optimal combination screening and core metabolite identification results. As shown in Figure 5A, the three-dimensional comparison clearly identified the optimal combination: saliva samples at 17 days post-insemination (dpi) using the RF model, which achieved the highest accuracy and most stable performance across both training and test sets. Figure 5B further revealed that RF model feature importance for saliva metabolites remained consistent across 12–18 dpi, with glucose (Glc), steroids (Ste), and xanthine-hypoxanthine (Xan) as the core contributors. During sequential feature reduction (Figure 5C,D), removing the first 3 metabolites had minimal impact on accuracy, but removing more than 4 metabolites led to a significant decline in performance, confirming that the top 4 metabolites are essential for accurate diagnosis. These results validated the superiority of saliva-based RF models at Day 17 and identified the core metabolite biomarkers. Comprehensive analysis revealed that saliva samples exhibited relatively stable predictive performance for sow pregnancy diagnosis across different gestational days and model types. The RF model not only demonstrated excellent fitting ability in the training sets of different samples but also exhibited outstanding predictive performance for the test set samples. Regarding the predictive performance of the RF model on saliva samples, 17 days post-insemination was identified as the earliest time point to achieve optimal predictive performance. Therefore, this study designated sow saliva samples at 17 days post-insemination as the target samples for early sow pregnancy diagnosis and selected the RF model as the primary model for early sow pregnancy diagnosis (Figure 5A). The selection of day 17 is biologically meaningful: in pigs, the conceptus undergoes rapid elongation and attachment between days 12 and 18, with maternal recognition of pregnancy established by day 14–15. By day 17, the placenta is sufficiently developed to produce a stable and detectable metabolic signature in maternal saliva, while still allowing early intervention (e.g., re-breeding non-pregnant sows) to minimize non-productive days.

### 3.4. The Optimal Model (RF) Reveals the Core Diagnostic Metabolite Features

In the RF model, the mean impurity decrease method was employed to evaluate the importance of different metabolites in the model. The results showed that the importance of certain metabolites in saliva samples at different gestational ages exhibited significant dynamic variations, such as phenols, primary amines, and glucose, whereas the importance of other metabolites remained stable. In saliva samples collected at 17 days post-insemination, the metabolites were ranked by importance from lowest to highest as follows: flavonoids (5.4%), primary amines (11.4%), phenols (12.1%), phosphate (13.2%), xanthine/hypoxanthine (17.9%), steroids (19%), and glucose (21.1%) (Figure 5B).

### 3.5. High-Precision and Streamlined Diagnostic Model Based on Feature Importance Optimization

After obtaining the weights of various metabolites in the RF model for sow saliva samples at 17 days post-insemination, this study employed the recursive feature elimination (RFE) method for stepwise optimization: first, the metabolite with the lowest weight was removed from the model, and the remaining metabolites were used to retrain the RF model; subsequently, the aforementioned “elimination-retraining” process was repeated, and the prediction accuracy of the RF model in each training round was recorded. The experimental results indicated that the predictive performance of the model remained generally stable as the number of eliminated metabolites increased from 0 to 4. However, when the number of eliminated metabolites reached 5 (with only 2 metabolites retained), both the fitting effect and predictive performance of the model decreased significantly, failing to maintain a stable, high-precision prediction level. It can be concluded that the minimum number of metabolites required to achieve optimal prediction results was three, namely Glc, Ste, and Xan (Figure 5C,D). Glucose is the primary energy substrate for the embryo and placenta; its high importance likely reflects the increased glucose demand during rapid conceptus growth. Steroids (predominantly progesterone and oestrogens) are key regulators of uterine receptivity and immune tolerance. Xanthine/hypoxanthine, as purine metabolites, may be linked to trophoblast proliferation and oxidative stress adaptation. The stable importance of these three metabolites across 17 dpi suggests they are robust biomarkers independent of exact sampling day.

### 3.6. Validation: The Streamlined Diagnostic Protocol Based on the Core Feature Combination Demonstrates Excellent Efficacy

**Figure 6:**
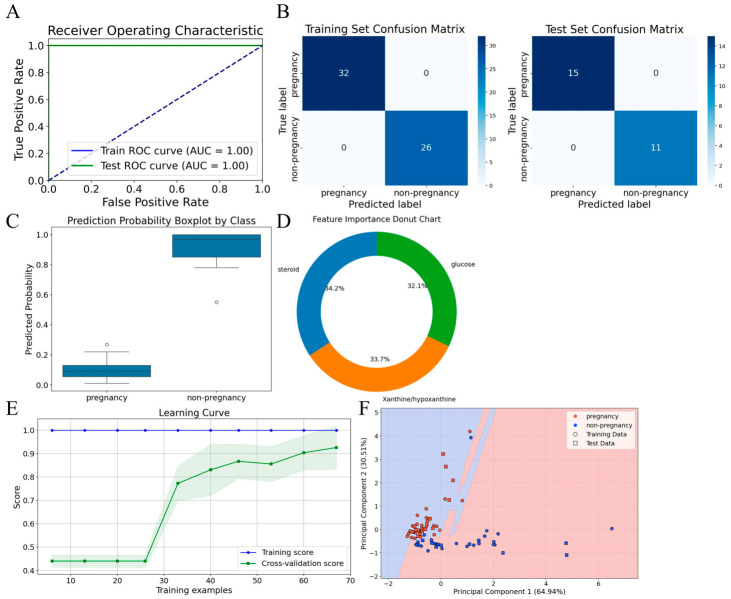
Comprehensive performance evaluation of the optimal RF model for sow pregnancy diagnosis using Saliva metabolites at 17 Days post-insemination (dpi). (**A**) Receiver Operating Characteristic (ROC) curves for the training and test sets, with an Area Under the Curve (AUC) of 1.00 for both sets, indicating perfect classification performance. (**B**) Confusion matrices for the training (*n* = 58 samples) and test (*n* = 26 samples) set, showing the number of true vs. predicted labels for pregnancy and non-pregnancy status. (**C**) Box Plots of the predicted probabilities for pregnancy and non-pregnancy classes, demonstrating distinct separation between the two groups. (**D**) Feature importance donut chart, illustrating the relative contribution of three core metabolites: glucose (32.1%), xanthine/hypoxanthine (33.7%), and steroids (34.2%). (**E**) Learning curves showing the training score and cross-validation score as a function of training examples. The green shaded area represents the 95% confidence interval. (**F**) Model decision boundaries visualized via Principal Component Analysis (PCA), with the red background indicating the prediction region for pregnancy and the blue background for non-pregnancy. Principal Component 1 (PC1, 64.94%) and Principal Component 2 (PC2, 30.52%) explain the majority of data variance.

After identifying the three core metabolites (glucose, steroids, and xanthine-hypoxanthine) from the RFE optimization, their concentration data from saliva samples at 17 dpi were used to retrain the RF model. Figure 6 validates the superior performance of this parsimonious three-metabolite model.

As shown in Figure 6A, the RF model achieved a perfect AUC score of 1.00 on both the training and test sets, indicating zero misclassification. The confusion matrix (Figure 6B) confirmed this accuracy: all 58 training samples (32 pregnant, 26 non-pregnant) and 26 test samples (15 pregnant, 11 non-pregnant) were correctly classified. Figure 6C shows that predicted probabilities for pregnant samples were consistently close to 1.0, while those for non-pregnant samples were close to 0, with no overlap. Feature importance analysis (Figure 6D) revealed a balanced contribution from the three metabolites: xanthine-hypoxanthine (33.7%), steroids (34.2%), and glucose (32.1%). The learning curves (Figure 6E) demonstrated rapid convergence and no overfitting: the cross-validation score increased sharply once the training sample size reached 30 and eventually converged to the training set score. Finally, the PCA-based decision boundary plot (Figure 6F) clearly separated pregnant from non-pregnant samples, visually confirming the model’s strong discriminative ability.

Biologically, the excellent diagnostic performance of this three-metabolite combination can be explained by their distinct physiological roles in early pregnancy. Glucose is the primary energy substrate for the rapidly growing conceptus and placenta; its elevation in maternal saliva reflects increased glucose demand and maternal metabolic adaptation during implantation (days 12–18 in pigs). Steroids, predominantly progesterone and oestrogens, are key regulators of uterine receptivity, corpus luteum maintenance, and immune tolerance at the maternal-fetal interface. Xanthine and hypoxanthine are purine metabolites; their increased levels may be linked to enhanced nucleotide turnover in the trophoblast or a mild hypoxic state accompanying embryo attachment. The balanced weight of these three metabolites (each contributing approximately one-third) suggests that no single physiological axis dominates, and their combination captures the multi-faceted metabolic shift from a non-pregnant cyclic state to a gestational state.

Consistent with these findings, the precision, recall, and F1-score were all 1.00 for both training and test sets (Table 4), confirming that the RF model using glucose, steroids, and xanthine-hypoxanthine in saliva collected at 17 days post-insemination provides a highly accurate and reliable early pregnancy diagnosis in sows.

## 4. Discussion

Among the three body fluids (saliva, urine, vaginal secretions) and metabolite combinations evaluated, saliva samples collected on Day 17 post-insemination were confirmed to be the most ideal matrix for early pregnancy diagnosis in sows. In particular, the combined detection of Glc, Ste, and Xan, coupled with modeling using the RF algorithm, was significantly superior to other models, thereby providing an optimized solution for the accurate diagnosis of early pregnancy in sows (Table 5).

**Table 5:** Performance of Saliva-Based Diagnosis at 17 dpi Using Three Core Metabolites.

Item	Value
Optimal sample	Saliva at 17 dpi
Optimal model	Random Forest
Core biomarkers	Glc, Ste, Xan
Accuracy (train/test)	100%/100%
MCC	1.00
ROC-AUC	1.00
Advantages	Non-invasive; 5–8 days earlier than ultrasound; only 3 metabolites
Limitations	Requires laboratory detection; on-farm strip under development

### 4.1. Metabolite Concentration Characteristics in Different Body Fluids

By comparing the concentration changes in various metabolites across different post-insemination days and body fluid types, it was found that the differences in metabolite concentrations between pregnant and non-pregnant samples were tissue-specific. In saliva, except for glucose, the contents of all other metabolites were significantly lower in pregnant samples than in non-pregnant samples—this phenomenon is closely associated with the adaptive physiological regulation of sows during early pregnancy. During early pregnancy, the progesterone level in sows increases significantly to maintain the stability of the uterine environment, while the estrogen level decreases temporarily to prevent excessive uterine contractions. Given that saliva composition is highly correlated with blood components, the reduced steroid content in saliva at this stage can reflect the changes in blood estrogen levels [16,17]. Meanwhile, during early pregnancy, insulin and insulin-like growth factors are significantly upregulated under the regulation of carbohydrate metabolism, ensuring the preferential and targeted supply of nutrients to the embryos. This process activates the mTOR signaling pathway, inhibits the catabolism of the liver and skeletal muscles, and consequently leads to decreased levels of phosphate and purine metabolites involved in energy transport in saliva [18]. As precursors and metabolites of protein synthesis, primary amines are characterized by a bias of nitrogen metabolism toward the uterus during pregnancy. Consequently, primary amines in saliva are reabsorbed into the circulatory system, resulting in a reduction in their levels [19,20]. Flavonoids and phenols possess antioxidant activity. To protect embryos from excessive oxidative stress-induced damage during pregnancy, maternal sows may enhance hepatic metabolism, thereby reducing the levels of these two classes of substances in saliva [21,22,23]. However, except for days 14 and 18 post-insemination, glucose concentrations showed no significant difference between pregnant and non-pregnant samples on all other days. This observation may be attributed to the fact that glucose is an exogenous nutrient, whose primary source in the oral cavity of sows is feed rather than endogenous synthesis [24].

In urine, the differences in various metabolites between samples collected at different post-insemination days and non-pregnant samples exhibited temporal specificity: some metabolites (e.g., phenols, phosphates, primary amines) showed significant concentration differences compared with non-pregnant samples only at specific time points, whereas xanthine/hypoxanthine, flavonoids, and glucose displayed extremely significant concentration differences at all post-insemination days relative to non-pregnant samples. Among these, xanthine/hypoxanthine are purine metabolites (adenine and guanine are oxidized to form hypoxanthine, which is further oxidized to generate xanthine). Additionally, most xanthine and hypoxanthine derived from the diet are decomposed by intestinal flora, with only a small amount excreted into the urine of animals [25]. Flavonoids and glucose are both exogenous nutrients that are not synthesized by the animals themselves. The levels of these three classes of metabolites in the urine of pregnant sows were higher than those in non-pregnant sows, which may be associated with the metabolic adjustments of sows during early pregnancy to meet the developmental demands of embryos [26,27]. Specifically, excessive activation of xanthine oxidase leads to increased production of reactive oxygen species (ROS) and uric acid in sows, triggering oxidative stress and inflammatory responses; in contrast, the elevated progesterone expression in sows post-conception inhibits the expression and activity of xanthine oxidase [28,29,30]. Flavonoids are potent antioxidants. During the embryonic implantation stage of early pregnancy, a local inflammatory response occurs in the endometrium to facilitate embryonic adhesion, whereas excessive oxidative stress may induce embryonic cell damage [31]. Flavonoids can alleviate oxidative stress by scavenging free radicals, thereby protecting embryos. At this stage, the maternal body may enhance the intestinal absorption of diet-derived flavonoids to meet embryonic demands, resulting in the accumulation of flavonoids in the body and their subsequent excretion via urine [32]. Glucose is the core energy substance in animals. After sows become pregnant, the placenta begins to secrete hormones such as progesterone and estrogen, which reduce the sensitivity of maternal tissues to insulin and induce a mild elevation in blood glucose levels. When blood glucose exceeds the renal tubular reabsorption threshold, the unreabsorbed glucose is excreted in the urine [33]. Meanwhile, during pregnancy, renal blood flow and glomerular filtration rate in sows increase, leading to greater amounts of glucose being filtered into the primary urine and further elevating urinary glucose concentrations [34,35,36].

In vaginal secretions, most metabolites (e.g., phenols, steroids, xanthine/hypoxanthine, flavonoids, glucose) showed no significant concentration differences compared with non-pregnant samples on most days from 12 to 18 post-insemination. This observation may be attributed to the complex vaginal environment of sows, which harbors a diverse array of microbial communities, thereby rendering the metabolite concentrations in vaginal secretions highly susceptible to interference and instability [37]. The common microbial phyla in the sow vagina include Proteobacteria, Firmicutes, and Bacteroidetes [38,39,40]; these microbes can produce metabolites such as short-chain fatty acids, amino acid metabolites, lipid metabolites, and vitamins through their metabolic activities [41,42], which may interfere with the quantitative detection results of the target metabolites in vaginal secretions.

In addition, this study found that certain metabolites—including phenols, primary amines, steroids, xanthine/hypoxanthine, flavonoids, and glucose in saliva; phenols, phosphates, primary amines, xanthine/hypoxanthine, and glucose in urine; as well as phenols, phosphates, and steroids in vaginal secretions—all exhibited obvious concentration changes from 12 to 14 days post-insemination, whereas their concentrations tended to stabilize and showed no significant differences from 15 to 18 days post-insemination. This finding indicates that sows underwent a distinct transformation in their physiological state from 12 to 14 days post-insemination, which then gradually stabilized thereafter.

### 4.2. Comparison of Machine Learning Models for Pregnancy Diagnosis

After fitting metabolite characteristic data with various machine learning models, comparisons of the accuracy and reliability of pregnancy diagnosis across different body fluid samples and post-insemination days, as well as across each model, revealed the following: saliva samples yielded the best performance in pregnancy diagnosis, followed by urine samples—this outcome may be associated with changes in nutritional requirements and adjustments in metabolic stress in sows post-pregnancy [43]; in contrast, the concentrations of various metabolites in vaginal secretions fluctuated significantly, leading to poor pregnancy diagnosis performance across all models. Comparisons of model performance indicated that the RF model exhibited the optimal fitting degree for the training set and predictive performance for the test set across different samples, followed by the XGB model and the MLP model. This indicates that tree-based models (RF, XGB) can effectively capture the nonlinear relationships between features and perform efficient pattern recognition when processing high-dimensional data [44]. The NB model exhibited the worst overall performance, with its predictive reliability for one class of samples being significantly higher than that for the other. This phenomenon can be attributed to the core assumption of the NB model—namely, that features are mutually independent. When this assumption is not satisfied by the dataset, the model’s performance becomes limited [45]. The results of this study indirectly demonstrate that the seven types of metabolites in the body fluids of pregnant and non-pregnant sows are not mutually independent but exhibit complex interactions. Such interactions may reflect the systemic metabolic reprogramming that occurs in the maternal body during early pregnancy to support embryonic development, such as the synergistic regulation between hormone metabolites (steroids) and energy metabolites (glucose). Therefore, tree-based models, which are capable of handling complex interactions between features, achieved superior diagnostic performance in this study.

### 4.3. Feature Screening and Core Metabolite Identification

This study ultimately identified saliva samples from sows at 17 days post-insemination as the optimal samples for early pregnancy diagnosis, and the RF model as the preferred machine learning model. Feature importance was calculated using the mean impurity decrease method, which revealed that although glucose showed no significant concentration difference between pregnant and non-pregnant samples, it exhibited the highest feature importance weight. This apparent discrepancy—no significant difference in Glc concentration but high feature importance—arises because the random forest model captures nonlinear interactions between Glc, Ste, and Xan rather than relying on single-variable differences. This indicates that despite the absence of a significant difference in glucose concentration between pregnant and non-pregnant samples, glucose engages in important interactions with the other two key features: steroids and xanthine/hypoxanthine. The random forest model can accurately capture such nonlinear synergistic effects. Glucose alone cannot directly determine the pregnancy status of sows, but it can enhance the relationship between steroids, xanthine/hypoxanthine, and the pregnancy status of sows.

During the processes of model optimization and core feature screening, fixed-order feature reduction may inadvertently lead to the elimination of key features, whereas dynamically adjusted screening strategies are more likely to capture the interactive relationships among metabolites. Therefore, this study adopted an approach that dynamically removed the features with the lowest weights based on the metabolite feature weights of the current model after each round of reduction. The results of stepwise reduction revealed the following: After the first round of reduction (removing the flavonoid feature), the model’s accuracy on the test set decreased slightly; after the second round (removing the phosphate feature), the model’s performance remained unchanged; after the third round (removing the phenolic feature); the model’s performance improved, with the test set accuracy recovering to 100%; subsequent removal of the primary amines feature did not affect the model’s optimal performance. This indicates that, in the saliva of sows at 17 days post-insemination (the optimal time point identified in this study), flavonoids may act as synergistic features—they need to cooperate with other core metabolites to exert their information contribution effect, which is consistent with their main physiological function (antioxidant synergism) [46]. Phenols may serve as interfering features, as their presence can introduce noise into the model and reduce predictive performance. In contrast, phosphates and primary amines may be redundant features, as their removal does not significant impacting the model’s performance.

Ultimately, this study identified three classes of metabolites—glucose, steroids, and xanthine/hypoxanthine—as the core indicators for the early pregnancy diagnosis of sows. The RF model trained on these three metabolites exhibited excellent performance, with strong classification capability and generalization ability. This indicates that these three metabolites may serve as core physiological biomarkers directly associated with the pregnancy status of sows. Specifically, steroids are involved in the metabolic regulation of pregnancy-related hormones [47,48]; xanthine/hypoxanthine are closely associated with nucleic acid metabolism in sows during pregnancy [49]; and glucose reflects changes in the energy demands of sows throughout pregnancy.

### 4.4. Study Limitations and Future Research Directions

In this study, due to the small sample size and the high conception rate of sows at the experimental farm (only 2 out of 79 inseminated sows failed to conceive), additional non-inseminated sows were included as non-pregnant control samples. A study by Christenson et al. [50] demonstrated that around day 12 post-insemination, if no embryos or an insufficient number of embryos are present in the sow’s uterus, the uterus secretes prostaglandins to promote corpus luteum regression, which in turn prevents the sustained secretion of pregnancy-related hormones. Therefore, non-pregnant sows (those that failed to conceive post-insemination) are physiologically similar to non-inseminated sows and can thus be effectively distinguished from pregnant sows. Future studies will expand the sample size, increase the collection of post-insemination non-pregnant samples, and further verify the reliability and accuracy of the method proposed in this study.

Traditional methods for sow pregnancy diagnosis must be delayed until 22–25 days post-insemination; with the support of some state-of-the-art equipment and technologies, the timeline can be advanced to 20 days post-fertilization. However, the estrous cycle of non-pregnant sows is typically 21 days, which makes it highly prone to missing the optimal rebreeding window and consequently leads to increased feeding costs. The pregnancy diagnosis method established in this study—using saliva samples collected at 17 days post-insemination—offers the advantages of convenient sampling and high timeliness, thereby providing a novel technical approach for the intensive management of sows post-insemination.

Although the experimental methods currently employed in this study (such as high-speed centrifugation, PCR instrument incubation, microplate reader detection, etc.) ensure data accuracy, they are highly dependent on a professional laboratory environment, involve relatively high costs, and present obvious operational barriers—making them difficult to directly apply to the rapid detection requirements of production sites. Therefore, in future research, we will focus on developing portable detection technologies suitable for on-site applications: First, we will attempt to use a small vacuum filtration device to replace the centrifuge, simplifying the pretreatment process of saliva samples; second, based on the three key metabolic markers identified in this study (glucose, steroids, and xanthine/hypoxanthine), we will develop lateral flow test strips, portable electrochemical sensors, or surface-enhanced Raman spectroscopy (SERS) detection platforms capable of simultaneously detecting these three substances; finally, the trained RF model will be integrated into a smartphone application, enabling breeders to directly input detection readings and obtain real-time diagnostic results for sow pregnancy status, thereby realizing the transformation of laboratory technology into practical tools for pig farms.

## 5. Conclusions

This study establishes a novel method for the early pregnancy diagnosis of sows by integrating chemical detection techniques with machine learning modeling. Using chemical colorimetry, the concentrations of seven types of metabolites in three bodily fluids were quantitatively detected in sows at 12–18 days post-insemination. Based on the detection data, seven machine learning models were trained, and the accuracy and reliability of these seven models for sow pregnancy diagnosis across different bodily fluids and time points were evaluated using the test set. The results indicated that saliva samples were the most ideal specimens for sow pregnancy diagnosis, while the RF model exhibited superior comprehensive performance compared with other models. Moreover, the diagnostic capability of samples from different bodily fluids and groups for pregnant sows improved with increasing days post-insemination. Finally, this study determined that saliva samples collected on day 17 post-insemination in sows, combined with the RF model, constitute the optimal method for the early pregnancy diagnosis of sows. Based on the feature importance analysis of the model, three core metabolites—glucose, steroids, and xanthine/hypoxanthine—were further identified, which significantly simplified the detection process while ensuring diagnostic efficacy. Building on the findings of this study, we will further develop a practical method for the early pregnancy diagnosis of sows.

## Figures and Tables

**Table 1:** Metabolic Fluid Samples (Saliva, Urine, Vaginal Secretion).

	NP *	P12 *	P13	P14	P15	P16	P17	P18	All
Saliva	35	44	44	49	46	49	49	51	367
Urine	35	41	42	43	48	48	43	40	340
Vaginal secretion	30	53	47	43	45	43	40	51	352
All	100	138	133	135	139	140	132	142	1059

*: P12 to P18 refer to body fluid samples collected from sows 12 to 18 days after insemination, while NP represents body fluid samples from non-mated or unsuccessful insemination sows in the same batch.

**Table 2:** Hyperparameters of Seven Machine Learning Models.

Model	Core Hyperparameters	Value/Configuration
Multilayer Perceptron	Number of hidden layers	1
	The number of neurons in each hidden layer	100
	activation function	Tanh (hidden layers) Softmax (outputlayers)
	learning rate	0.001
	optimizer	adam
	Epochs	200
Random Forest	n_estimators	100
	max_depth	None
	max_features	auto
	min_samples_leaf	1
Naive Bayes	var_smoothing	1 × 10^−9^
	prior probability distribution	None
Logistic Regression	Regularization coefficient	1.0
	Regularization type	Ridge
	solver	1bfgs
Fisher Linear Discriminant	shrinkage	None
	Type of covariance matrix	pooled covariance matrix
Support Vector Machine	Regularization coefficient	1.0
	Kernel function type	linear
	Kernel function parameters	scale
Extreme Gradient Boosting	learning_rate	0.1
	n_estimators	100
	max_depth	6
	Regularization type	Ridge
	objective	binary:logistic
	Regularization coefficient	lambda

**Table 3:** Differential metabolites related to early pregnancy in different body fluids.

Body Fluid	Target Chemical Substance	Variation Trend (Pregnancy vs. Non-Pregnancy)	Key Time Point
Saliva	Pheo/Pho/Ami/Ste/Xan/Fla	Down	14d
	Glc	No significant difference	All times
Urine	Pheo/Pho/Ste/Xan/Fla/Glc	Up	14d
	Ami	First increase and then decrease.	14d
Vaginal Secretion	Pheo/Pho/Ami/Ste	The initial decline was followed by no significant difference.	A specific point in time
	Xan/Fla/Glc	no significant difference	All times

**Table 4:** Performance parameters of the random forest model for pregnancy diagnosis based on three metabolites in saliva samples of sows 17 days after insemination.

		Precision	Recall	F1-Score	Support
**Training Set**	Pregnancy	1.00	1.00	1.00	32
	Non-pregnancy	1.00	1.00	1.00	26
	accuracy			1.00	58
	macro avg	1.00	1.00	1.00	58
	weighted avg	1.00	1.00	1.00	58
**Test Set**	Pregnancy	1.00	1.00	1.00	15
	Non-pregnancy	1.00	1.00	1.00	11
	accuracy			1.00	26
	macro avg	1.00	1.00	1.00	26
	weighted avg	1.00	1.00	1.00	26

## Data Availability

The data presented in this study are available on request from the corresponding author due to protect the intellectual property rights of this study.

## Supplementary Materials

The following supporting information can be downloaded at: https://www.mdpi.com/article/10.3390/vetsci13050409/s1. Figure S1: Standard curves of concentration versus absorbance for the detection of seven metabolite categories; Figure S2: Changes in the concentrations of various metabolite molecules with soaking time of vaginal swabs in 60% anhydrous ethanol.

## Abbreviations

The following abbreviations are used in this manuscript:

Ami	Primary amines
Fla	Flavonoid
FLD	Fisher Linear Discrimination
Glc	Glucose
LR	Logistic Regression
MCC	Matthews Correlation Coefficient
MLP	multilayer perceptron
NB	Naive Bayes
NP	Non-pregnant sow
P	Pregnant sow
Pheo	Phenols
Pho	Phosphates
RF	Random Forest
RFE	Recursive Feature Elimination
ROC_AUC	Area Under the Receiver Operating Characteristic Curve
Ste	Steroids
SVM	Support Vector Machine
Xan	Xanthine/Hypoxanthine
XGB	XGBoost
