# Supplementary material for: Study on Early Pregnancy Diagnosis of Sows Based on Body Fluid Metabolite Detection Combined with Machine Learning Models
Source: Vet Sci. 2026 Apr 22;13(5):409. doi: 10.3390/vetsci13050409 (PMC13211673; doi:10.3390/vetsci13050409)
Supplement: Supplementary file 1 [file vetsci-13-00409-s001.zip › vetsci-4166242-supplementary.pdf]

veterinary sciences

MDPI

Supplementary Materials:

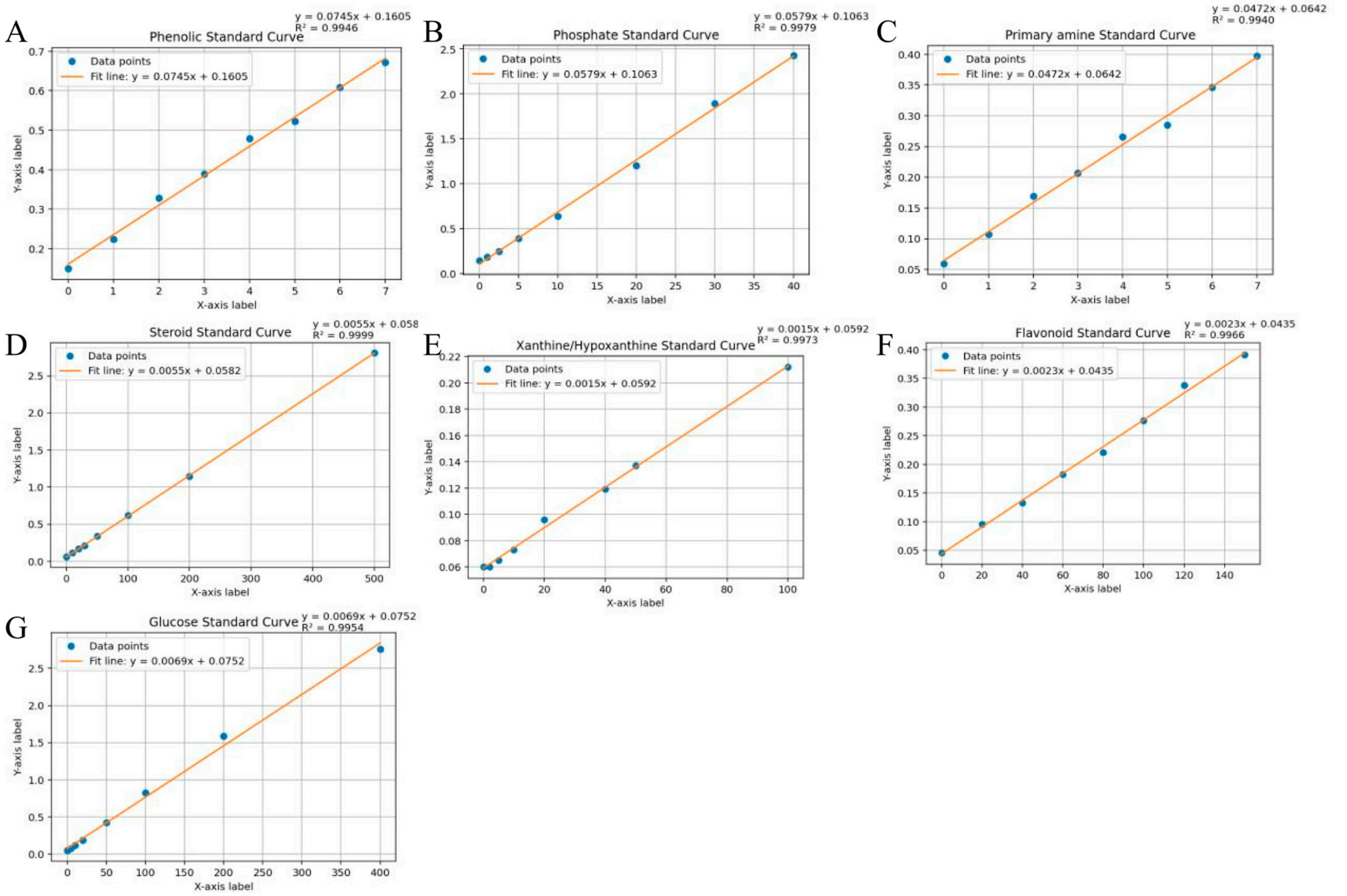

Figure S1 Standard curves of concentration versus absorbance for the detection of seven metabolite categories.(A) Standard curve for phenol detection.(B) Standard curve for phosphate detection.(C) Standard curve for primary amine detection.(D) Standard curve for steroid detection.(E) Standard curve for xanthine/hypoxanthine detection.(F) Standard curve for flavonoid detection.(G) Standard curve for glucose detection.

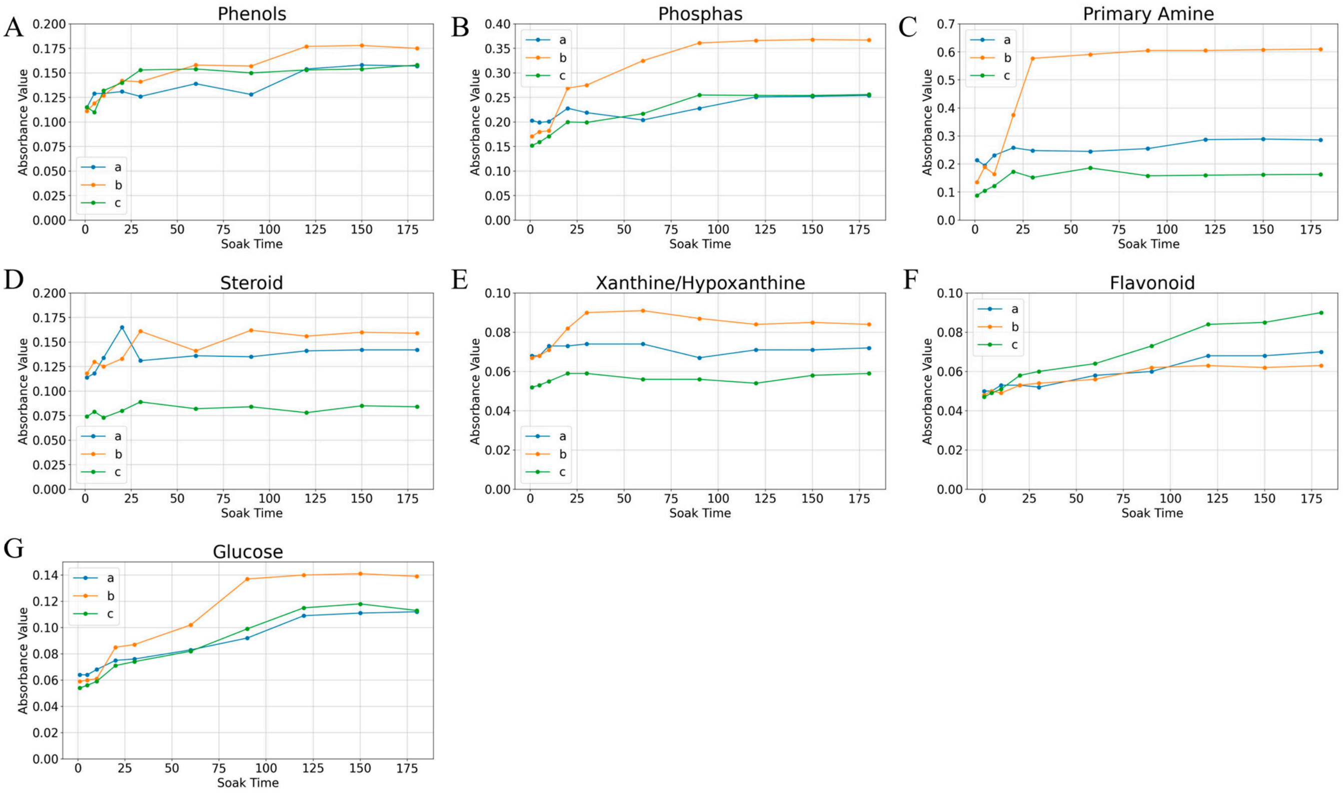

Figure S2 Changes in the concentrations of various metabolite molecules with soaking time of vaginal swabs in 60% anhydrous ethanol.(A) Changes in phenol concentrations with time.(B) Changes in phosphate concentrations with time.(C) Changes in primary amine concentrations with time.(D) Changes in steroid concentrations with time.(E) Changes in xanthine/hypoxanthine concentrations with time.(F) Changes in flavonoid concentrations with time.(G) Changes in glucose concentrations with time.
